# Acute ethmoiditis complicated by intraorbital abscess, orbital cellulitis, and cerebral empyema in a 14‐year‐old girl

**DOI:** 10.1002/ccr3.6984

**Published:** 2023-02-24

**Authors:** Moise Mbaluku Colombe, Erick Heri Nabuloho, Fernand Manga Opondjo, Viviane Feza Bianga, Fikiri Bavurhe Rodrigue, Stéphanie Sifa Isonga, Peniel Kundo Shemahamba, Archippe Muhandule Birindwa

**Affiliations:** ^1^ Pediatrics Department University Clinics of Bukavu Bukavu Democratic Republic of the Congo; ^2^ Department of Pediatrics, Faculty of Medicine Official University of Bukavu Bukavu Democratic Republic of the Congo; ^3^ Ophthalmology Department University Clinics of Bukavu Bukavu Democratic Republic of the Congo; ^4^ Department of Infectious Diseases Institute of Biomedicine, University of Gothenburg Gothenburg Sweden; ^5^ Pediatrics Department of the Centre Hospitalier de l'Ouest Guyanais Franck Joly (Saint‐Laurent‐du‐Maroni) French Guyana France

**Keywords:** acute ethmoiditis, Bukavu, cerebral empyema, intraorbital abscess

## Abstract

We report the case of a 14‐year‐old girl with pain and protrusion of the left eye and treated with diclofenac. Clinical and paraclinical examinations revealed a cerebral empyema and a left retro‐orbital abscess complicating an acute ethmoiditis. Parenteral antibiotic remains essential in the management of acute ethmoiditis to prevent complications.

## Graphical abstract

Cerebral empyeme, retro‐orbital abscess were the complications found in our case. Situation having caused by the delay of diagnosis and adequate care. The early care remains the guarantee of a good outcome.

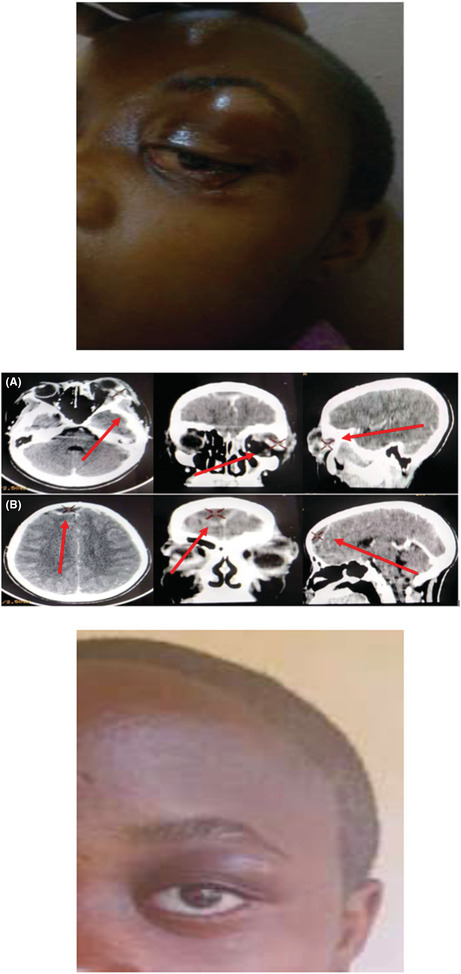

## 1 INTRODUCTION

Ethmoiditis is a frequent form of pediatric sinusitis and remains the most observed complication of rhinopharyngitis in children.^1^ It can occur at a very young age, the ethmoidal sinus being already well developed and separated from the nasal cavities at birth.^2^ During the first decade of life, acute sinusitis and especially ethmoiditis, are frequent. They represent 21% of all antibiotic prescriptions in pediatrics. Orbital involvement accounts for up to 91% of complications, with orbital and subperiosteal abscesses often leading to cavernous sinus thrombosis, meningitis, blindness, and intracranial abscesses.^3^, ^4^ Infection often spreads from the ethmoidal sinus into the periorbital spaces through the bloodstream or via erosion of the lamina papyracea.

When a subperiosteal orbital abscess (Chandler stage III) develops, surgical drainage is mandatory in addition to intravenous antibiotics.

We present a 14‐year‐old girl with acute ethmoiditis complicated by cerebral empyema, cellulitis, and intraorbital abscess.

Medical and surgical treatment resulted in a gradual and rapid resolution of symptoms while maintaining neurological and visual integrity.

## 2 PATIENT AND OBSERVATION

The patient was a 14‐year‐old girl living in the commune of Kadutu in the city of Bukavu. Her parents brought her to pediatric emergency room of the University Clinics of Bukavu for painful swelling and protrusion of the left eye, which had been developing for several days following nasopharyngitis. She was treated at home with a diclofenac tablet without success.

**FIGURE 1:**
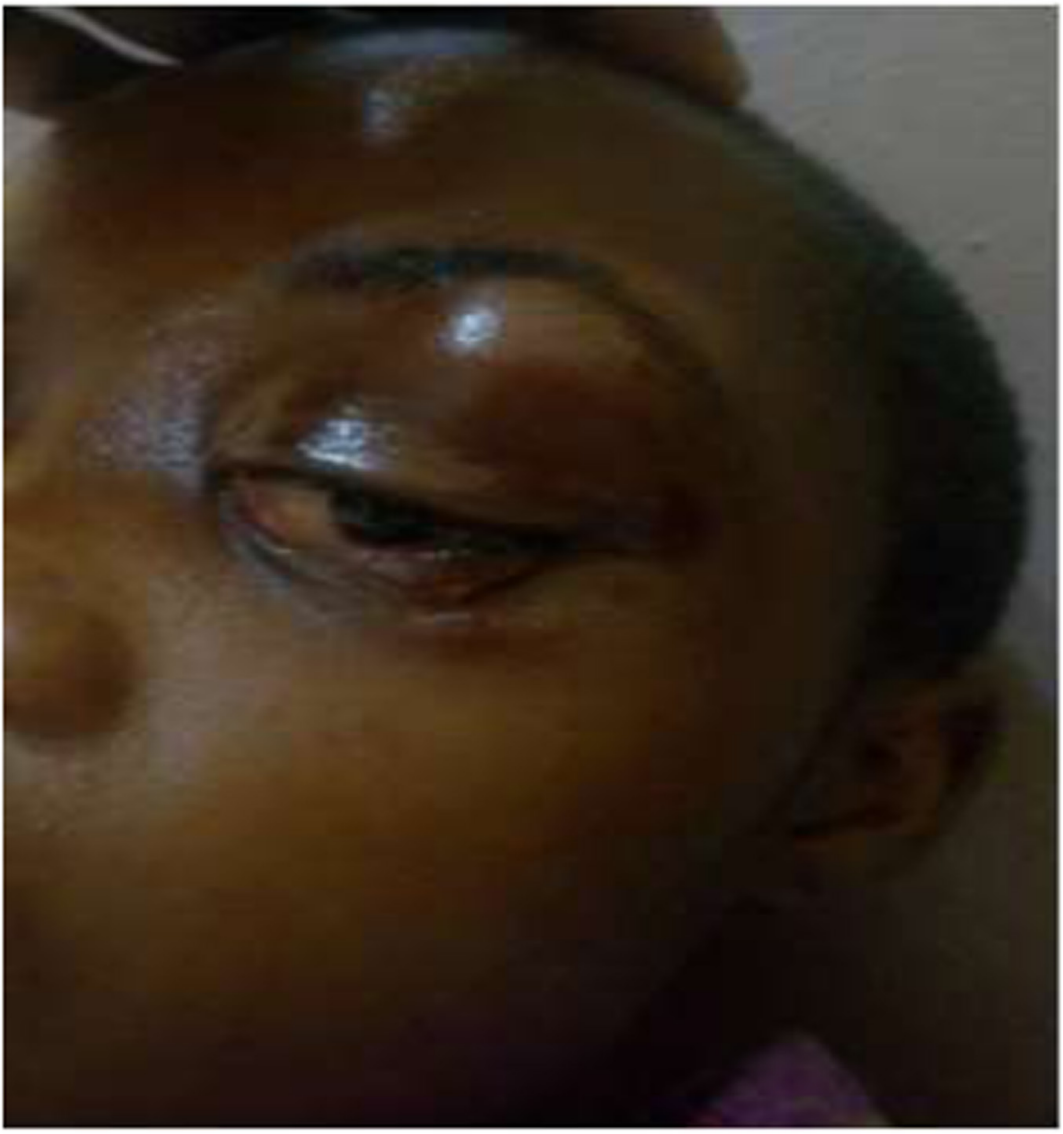
Image of the teenager's face showing the orbital abscess on the seventh day (image published with the consent of the child's parents).

The complementary history revealed headaches and a notion of a recurrent cold. The physical examination revealed a change in the general state due to a decrease in physical activity as well as a high fever of 39.9°C, a sensitive swelling of the left upper eyelid and the external canthus, chemosis, and left exophthalmos with preservation of ocular mobility and visual acuity (10/10) with a normal fundus and sensitivity to compression opposite the ethmoidal and maxillary sinuses (Figure 1).

**FIGURE 2:**
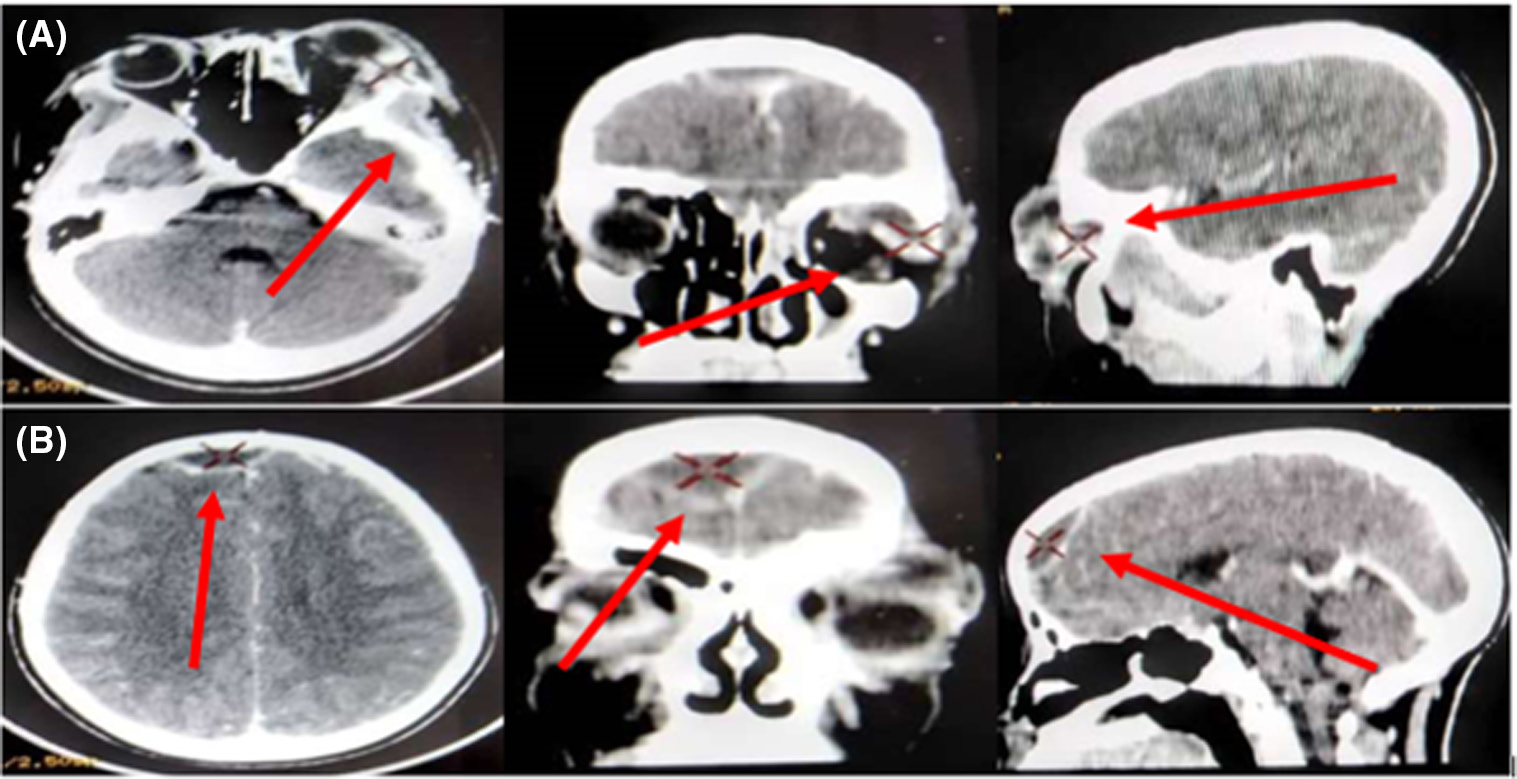
Images of the brain scan performed. (A) Images showing the left superior‐lateral intraorbital abscess. (B) Images showing the cerebral empyema.

The blood count revealed hyperleukocytosis at 17,700/mm^3^ with 93% neutrophilic predominance and significantly elevated inflammatory markers at 107 mg/L and 5.04 ng/mL, respectively, for CRP and Pro‐Calcitonin. Ethmoid‐orbital and brain CT scans revealed acute ethmoiditis with left maxillary sinus involvement complicated by a left superior‐lateral intraorbital abscess (22 × 13 × 12 mm) (Chandler Classification IV) (Figure 2) and a right‐predominant medial frontal brain empyema (25 × 14 × 10 mm) without involvement.

The diagnosis of acute ethmoiditis complicated by intraorbital abscess, orbital cellulitis, and cerebral empyema was made.

A parenteral treatment of ceftriaxone (100 mg/kg/day), ciprofloxacin (30 mg/kg/day) associated with a regressive dose of prednisone tablet at a rate of 0.5 mg/kg/day and an eye drop made of dexamethasone combined with ciprofloxacin was initiated. After 48 h of treatment, the evolution was marked by apyrexia and significant regression of headaches on the one hand, and by the aggravation of the swelling of the left eyelid, exophthalmos with the occurrence of exposure keratitis on the other hand. At 72 h of treatment, metronidazole infusion (30 mg/kg/day) and artificial tears were added to his treatment. On the seventh day, the orbital abscess was collected and drained under local anesthesia (Lidocaine) via an external route passing under the left superciliary arch. In pyoculture, no germs grew.

**FIGURE 3:**
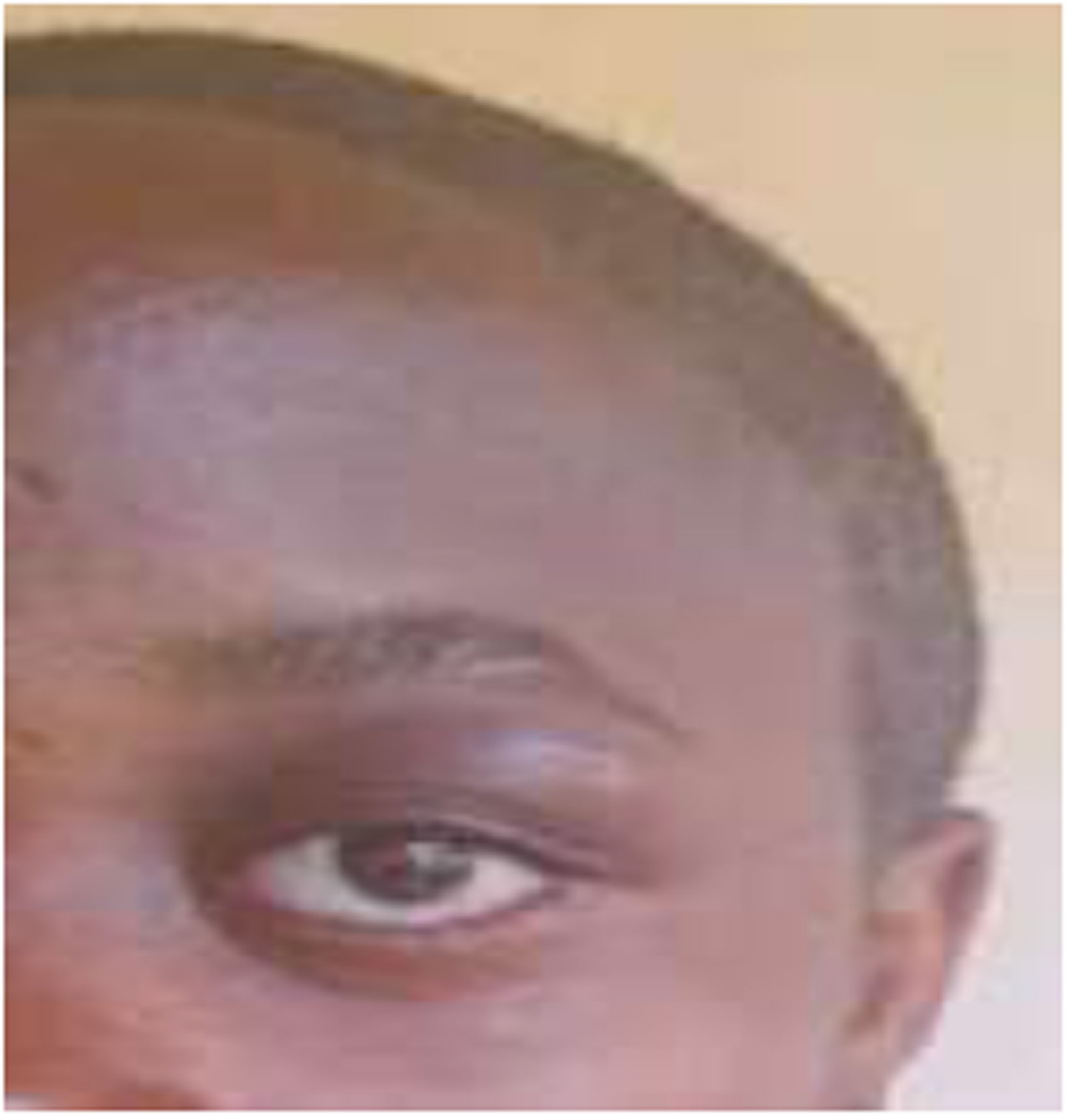
Image of the patient's face after drainage of the abscess (image published with the consent of the child's parents).

At 72 h after drainage of the abscess, we noticed a complete regression of the left exophthalmos, complete resorption of the bilateral swelling of the eyelids, and chemosis (Figure 3). On the 14th day of treatment, the control blood tests requested showed good paraclinical evolution with a decrease in the hyperleukocytosis as well as the initially elevated inflammation markers (Table 1). The patient was discharged under an oral treatment made of amoxicillin/clavulanic acid tablets (80 mg/kg/day), metronidazole tablets (30 mg/kg/day), and corticosteroid therapy (prednisone) at a regressive dose for 7 days.

**TABLE 1:** Table of blood tests performed.

At the entrance	First check at	Second control on day 24
**Blood cell count**	**Blood cell count**	**Blood cell count**
Leukocyte 17,000 elements/mm^3^	Leukocyte 15,000 elements/mm^3^	Leukocyte 7730 elements/mm^3^
Neutrophil 93% (15,810 elements/mm^3^)	Neutrophil 87% (elements/mm^3^)	Neutrophil 80.3% (6207 elements/mm^3^)
**Inflammation markers**	**Inflammation markers**	**Inflammation markers**
CRP 107 mg/L		
PCT 5.04 ng/mL	PCT <0.1 ng/mL	

## 3 DISCUSSION

Orbital involvement is the most frequent complication of acute ethmoiditis. Its frequency is about 91% of the complications of ethmoiditis in children, cerebral involvement being rarely observed.^4^ In this study, we report the case of an adolescent girl who presented with acute ethmoiditis complicated by cerebral empyema, orbital cellulitis, and intraorbital abscess. Acute ethmoiditis is generally secondary to bacterial superinfection of upper respiratory tract infection. Contrary to treatment with amoxicillin capsules alone or in combination with clavulanic acid in severe acute forms of maxillary rhinosinusitis, early parenteral antibiotic therapy in a hospital setting remains essential to prevent the potential complications of ethmoiditis that make it so serious: meningitis, empyema, abscesses, thrombophlebitis of the cavernous sinus or orbital complications.^5^ For this reason, a delay in hospital care can lead the child to develop the serious complications of the disease.

The clinic was dominated by a fever of 39.9°C, headache, a sensitive swelling of the left upper eyelid and the external canthus, chemosis, left exophthalmos with preservation of ocular mobility and visual acuity (10/10), a normal eye fundus with the notion of repeated rhinopharyngitis, and is in line with the literature which finds in the majority of cases similar symptomatology and rarely ophthalmoplegia and papilledema in cases of acute ethmoiditis in children.^4^, ^6^ A brain scan revealed acute ethmoiditis of the left maxillary sinus, complicated by right‐predominant medial frontal cerebral empyema and a left superior‐external intraorbital abscess (Chandler IV classification). The cerebral empyema observed in this case is consistent with data in the literature that supports the idea that children with upper orbital abscesses are more likely to have intracranial abscesses.^1^ The blood count revealed a hyperleukocytosis of 17,000 elements/mm^3^ with a neutrophilic predominance and an increase in CRP and Pro‐Calcitonin to 107 mg/L and 5.04 ng/mL, respectively. Several recent studies show that emergency ethmoid‐orbital and cerebral CT remain the examinations of choice for making the diagnosis and specifying the locoregional extension of the complications, allowing emergency treatment to be instituted. Our patient's clinical picture (high fever of 39.9°C, painful swelling, and protrusion of the left eye for several days the following nasopharyngitis) indicated this from the first day of hospitalization.^7^, ^8^, ^9^, ^10^, ^11^, ^12^ Inflammation with hyperleukocytosis remains an observation in several studies.^6^, ^13^ According to Chandler, most of the authors recommend empirical broad‐spectrum antibiotic therapy by the intravenous route, combining third generation cephalosporins or amoxicillin/clavulanic acid with quinolones, aminoglycosides, and imidazoles, depending on the stage of the disease.^1^, ^6^, ^14^, ^15^

While others recommend the combination of third generation cephalosporins and anti‐staphylococci as they support the idea that children under 10 years of age are more likely to be infected with *Streptococcus pneumoniae* or *Staphylococcus aureus* and those over 10 years of age are more likely to be infected with polymicrobial pathogens.^3^, ^13^, ^16^ In this case, antibiotic therapy with ceftriaxone, ciprofloxacin, gentamycin, metronidazole, and drainage of the intraorbital abscess were used. Orbital and subperiosteal abscesses, as well as orbital cellulitis, are the complications of ethmoidomaxillary sinusitis found by several authors,^2^, ^6^, ^17^ but in this study, the medial frontal cerebral empyema and focal signs were found beyond the orbital cellulitis and the Chandler IV retro‐orbital abscess, and this would be explained by the indirect propagation of the infection by the hematogenous route through the ophthalmic veins without valves. In our study, blood culture before starting antibiotic therapy and the search for soluble antigens would have increased the probability of identifying the germ in our study.^7^, ^18^

## 4 CONCLUSION

In children, the most serious complications of acute ethmoiditis are superior‐lateral intraorbital abscess, orbital cellulitis, and cerebral empyema. Fever, headache, palpebral edema, chemosis, and exophthalmos are common clinical manifestations. However, ethmoid‐orbital and cerebral CT scans continue to be used as a paraclinical means of definitive diagnosis and determining the locoregional extension. Adequate antibiotic therapy, possibly in conjunction with orbital abscess drainage, is a critical component of treatment.

## AUTHOR CONTRIBUTIONS

**Moise Mbaluku Colombe:** Writing – original draft. **Erick Heri Nabuloho:** Investigation. **Fernand Manga Opondjo:** Writing – review and editing. **Viviane Feza Bianga:** Writing – review and editing. **Fikiri Bavurhe Rodrigue:** Funding acquisition. **Stéphanie Sifa Isonga:** Resources. **Peniel Kundo Shemahamba:** Writing – original draft. **Archippe Muhandule Birindwa:** Supervision.

## FUNDING INFORMATION

None.

## CONFLICT OF INTEREST STATEMENT

The authors declare any conflicts of interest.

## PARENTAL CONSENT

Obtained.

## CONSENT

Written consent was obtained from the parents as the patient was under 16 years of age.

## Data Availability

All materials used in this study are available on request.
